# Tumor microenvironment and clinical efficacy of first line immunotherapy-based combinations in metastatic renal cell carcinoma

**DOI:** 10.1007/s12032-024-02370-0

**Published:** 2024-05-13

**Authors:** Enrico Sammarco, Martina Rossetti, Alessia Salfi, Adele Bonato, Paolo Viacava, Gianluca Masi, Luca Galli, Pinuccia Faviana

**Affiliations:** 1 Medical Oncology Unit, Livorno Hospital, Azienda Toscana Nord Ovest, Livorno, Italy; 2 Department of Surgical, Medical, Molecular Pathology and Critical Area, University of Pisa, Pisa, Italy; 3 Medical Oncology Unit 2, Santa Chiara Hospital, Azienda Ospedaliero-Universitaria Pisana, Pisa, Italy; 4 Pathology Unit, Livorno Hospital, Azienda Toscana Nord Ovest, Livorno, Italy; 5 Department of Translational Research and New Technologies in Medicine and Surgery, University of Pisa, Pisa, Italy

**Keywords:** Renal cell carcinoma, Tumor microenvironment, CD163, Immune checkpoint inhibitor

## Abstract

The impact of tumor microenvironment (TME) in influencing clinical response to first-line immune checkpoint inhibitor (ICI)-based treatment in advanced renal cell carcinoma (RCC) is unclear. Immunohistochemistry (IHC) could identify biomarkers related to immune checkpoints and immune cell population. This study retrospectively characterized TME from 28 RCC patients who received first line ICI-based therapy through IHC assessment of selected markers and explored preliminary evidence about their possible correlation with treatment efficacy. We found a significantly higher count of CD80+, CD163+ cells and their ratio in RCC with clear cell component compared to those without clear cell features; additionally, patients with metastatic disease at diagnosis were associated with higher expression of CD163+ cells, while higher count of CD4+ cells and CD4+/CD8+ ratio were found in RCC with sarcomatoid features. Patients achieving partial or complete response were associated with lower expression of CD163+ cells (median 28 vs 47; *p* = 0.049). Furthermore, lower expression of CD163+ was associated with better PFS (median PFS 20.0 vs 4.7 months; HR 0.22 *p* = 0.011) and OS (median OS NR vs 14.4 months; HR 0.28 *p* = 0.036). A longer OS was reported in PD-L1 CPS negative patients (median OS NR vs 11.8 months; HR 0.20 *p* = 0.024). High infiltration of CD163+ macrophages, who typically present “anti-inflammatory” M2-like phenotype, could identify a subgroup of patients with poor survival after receiving first-line ICI.

**Supplementary Information:**

The online version contains supplementary material available at 10.1007/s12032-024-02370-0.

## Introduction

The therapeutic landscape of metastatic renal cell carcinoma (RCC) has been dramatically revolutionized by the advent of immune checkpoint inhibitors (ICIs): immunotherapy-based combinations represent the current standard of care in first line treatment for RCC patients [1]. Dual checkpoint blockade (nivolumab plus ipilimumab, for patients with intermediate or poor risk disease according to International Metastatic RCC Database Consortium (IMDC) risk score [2]) and association of ICI and anti-angiogenic tyrosine kinase inhibitor (pembrolizumab plus axitinib [3], nivolumab plus cabozantinib [4], pembrolizumab plus lenvatinib [5], regardless of risk score) demonstrated overall survival improvement compared to sunitinib monotherapy in previously untreated advanced RCC patients [6].

Despite a clear survival benefit, most patients receiving an ICI-based first-line treatment develop disease progression, requiring the choice of a new systemic treatment; additionally, up to 20% of patients are refractory to combination treatments, rapidly developing disease progression [7]. To date, the IMDC score represents the main tool to provide prognostic information and to predict response to first line combination treatments; additional biomarkers that can predict response to ICI in first line of RCC are under investigation, but none are approved for use in clinical practice [8, 9].

Recently, more attention has been given to the role of the tumor microenvironment (TME) in modulating the response to systemic therapies [10]. In particular, due to the high density of immune cells (such as T cells and tumor-associated macrophages, TAMs), the evaluation of TME in RCC samples could represent a simple and useful assessment to identify potential biomarkers of response to ICIs [11, 12]. Detection by immunohistochemistry (IHC) of cluster of differentiation (CD) related to immune checkpoints and immune cells represents a well-established, readily available and cost-effective method to study TME composition [13, 14].

Therefore, the purpose of our study is to provide pathological characterization through immunohistochemical evaluation of selected markers of TME in RCC tumor samples from patients who had received immune-based combination therapy as first-line treatment for advanced disease and to find preliminary evidence about their possible correlation with clinical efficacy and activity of these treatments.

## Materials and methods

### Patient selection and data collection

Patients with histologically confirmed diagnosis of unresectable locally advanced or metastatic RCC who were treated with a first line immunotherapy-based combination treatment at Pisa oncology centre between June 2019 and June 2023 were retrospectively selected. All enrolled patients were treated according to current guidelines of European Society of Medical Oncology (ESMO) [1]. Archival formalin-fixed, paraffin-embedded (FFPE) samples of primary tumor or metastatic site obtained at diagnosis were retrieved from the pathology department.

The main inclusion criteria were: histological diagnosis of RCC; presence of advanced or metastatic (synchronous or metachronous) disease; the availability of sufficient histological material to perform an adequate IHC evaluation of TME; at least one cycle of a first-line combination treatment (including dual immune checkpoint blockade or ICI in combination with a TKI); at least one disease assessment to evaluate response to first-line therapy. In case of metachronous metastatic disease, histological material from a maximum of 3 months before the diagnosis of metastatic stage was required to be included. Patients without clear cell component were also included. A dedicated pathologist assessed the adequacy of material from FFPE tumor blocks.

For each patient, pathological data (stage at diagnosis, histology of RCC, nucleolar grading, presence of sarcomatoid features) and clinical data (previous nephrectomy, performance status, IMDC risk score, sites of metastasis) have been collected. In addition, data related to first-line immunotherapy-based treatment (type of combination therapy, best overall response and objective response rate-ORR, progression-free survival-PFS, overall survival-OS) have been recorded.

All information regarding human material was managed using anonymous codes and all samples were handled in compliance with the Declaration of Helsinki.

### Immunohistochemical analysis

A pathological assessment was performed on 5 µm sections from tumor samples, evaluating immunohistochemical expression of selected markers representative of tumor cell (TC) and immune cell (IC) population; a quantitative analysis of the expression of each marker has been reported. The number of positive cells was determined by scoring a minimum of 10 high-power fields (magnification, 40×) and counting the number of immunoreactive cells out of the total epithelial cells analyzed in each field of view.

Object counting was performed using the automatic image analysis tool of the *NIS Elements BR* digital analysis system (*NIS Elements BR, Basic Research, Vers. 5.20* Nikon Europe B.V., Stroombaan 14, Amstelveen, Netherlands) which allows the operator to create, through thresholds based on intensity values, binary levels able to recognize the different elements to be calculated. After having selected the high-power field on the optic microscope, the “auto-measurement” tool of the software calculated the number of objects in the area following the set-up values. The automated system detected the similar objects on the image and highlighted them by colour. The results were exported as an excel file containing a table that shows parameters such as the total area (e.g. 43,334,01 μm^2^ for each high-power field), the surface fraction and the number of detected objects for each evaluated field. Moreover, *NIS-Elements BR* calculated the basic statistics (e.g. mean value). Having carried out the quantitative analysis using the described software has made the evaluation operator-independent, generating a reliable and reproducible methodology.

The qualitative detection of PD-L1 (CD274) was assessed using Ventana PD-L1 (SP263) assay; the expression of PD-L1 in both TCs and tumor-infiltrating ICs was determined and was reported using combined positive score (CPS).

Immunophenotypic study of TME included assessment of expression of CD8+ and CD4+ T cell infiltration and the relative ratio. Macrophage population was assessed through IHC determination of CD163, a scavenger receptor that is highly expressed in TAMs with M2-like phenotype; this subgroup of macrophages is involved in downregulation of T cell function [15]. In addition, expression of CD80, generally considered a typical marker of activated macrophages with M1 features (with antitumor activity and involved in restoring an immune responsive microenvironment), was evaluated [16].

### Outcome evaluation and statistical analysis

Descriptive statistics were provided as frequency (rate) for categorical data and as median value and ranges for continuous variables. The chi-square test (or the Fisher’s exact test, when appropriate) was used to compare categorical data, while the Student’s t test and the Mann–Whitney test (or the Kruskal–Wallis test) were performed to compare means and medians of continuous data, respectively.

Survival times were calculated using the Kaplan–Meier method; Cox regression analysis was performed to find difference in clinical outcome. Statistical significance was defined as *p* < 0.05.

ORR was defined as the percentage of patients who achieve a response, which could either be complete response or partial response according to RECIST, version 1.1. PFS was defined as the time from the beginning of first-line therapy to the development of disease progression or death for any cause, whereas OS was calculated as the time from the beginning of first-line therapy to death for any cause or last follow-up.

Selected TME markers were evaluated according to the median value and interquartile range (IQR); the receiver operating curves (ROCs) method was performed to find reliable cut-off value for each marker. In case a reliable value could not be determined via ROCs, an arbitrary value (coinciding with the median value) was chosen for each marker and was used to calculate PFS and OS, comparing as two different groups.

Statistical analyses were performed using IBM® SPSS® Statistics version 26.0 (IBM Corp., Armonk, NY, USA).

## Results

### Patient characteristics

A total of 28 patients with advanced or metastatic RCC were retrospectively identified and stained for IHC analysis. Most patients were male and had a clear cell component (85.7%); sarcomatoid features were reported in 21.4% of patients. Most patients underwent previous nephrectomy (85.7%). According to IMDC score, the majority of patients had intermediate risk (78.6%). The most common site of metastasis was lung (67.9%), followed by lymph node (46.4%), bone (25%) and liver (17.9%). Overall, 12 patients received dual ICI as first-line treatment (nivolumab plus ipilimumab), whereas 16 received ICI in combination with TKI (12 pembrolizumab plus axitinib, 4 nivolumab plus cabozantinib). Baseline demographic and disease characteristics are reported in Table 1.

**Table 1:** Summary of baseline demographic, clinical and disease characteristics of enrolled patients

Characteristic	Number of patients (%)
Age, median (range)	59 (43–77)
Gender	
Male	23 (82.2)
Female	5 (17.8)
ECOG performance status	
0	18 (64.3)
1	8 (28.6)
2	2 (7.1)
Histology	
ccRCC	24 (85.7)
nccRCC	4 (14.3)
Nucleolar grading	
1–2	6 (21.4)
3–4	17 (60.7)
Unknown	5 (17.9)
Sarcomatoid features	
Yes	6 (21.4)
No	22 (78.6)
Nephrectomy	
Yes	24 (85.7)
No	4 (14.3)
Metastatic stage at initial diagnosis	
Yes	7 (25.0)
No	21 (75.0)
IMDC score	
Favorable	3 (10.7)
Intermediate	22 (78.6)
Poor	3 (10.7)
Most common sites of metastasis	
Lung	19 (67.9)
Lymph node	13 (46.4)
Bone	7 (25.0)
Liver	5 (17.9)
Adrenal gland	2 (7.1)
Subcutaneous/soft tissue	2 (7.1)
Type of 1st line combination treatment	
Dual ICI (nivolumab + ipilimumab)	12 (42.9)
ICI + TKI	16 (57.1)
Pembrolizumab + axitinib	12 (42.9)
Nivolumab + cabozantinib	4 (14.2)
Type of tumor sample	
Primary tumor	24 (85.7)
Metastasis	4 (14.3)

*ccRCC* clear cell RCC, *nccRCC* non-clear cell RCC

### TME assessment and clinicopathological features

The overall results of IHC assessment of selected TME markers from tumor samples are shown in Table 2. PD-L1 expression according to CPS score was negative in 20 patients (71.4%).

**Table 2:** Results of immunohistochemical assessment of TME in the entire population

TME marker	All patients (*N* = 28)
CD4+ IC	
Median (IQR)	23 (13–41)
CD8+ IC	
Median (IQR)	19 (7–25)
CD4+/CD8+ ratio, Median (IQR)	1.85 (0.8–2.78)
> 1 (%)	10 (35.7)
≤ 1 (%)	18 (64.3)
CD80+ IC	
Median (IQR)	88 (68–96)
CD163+ IC	
Median (IQR)	44 (27–56)
CD80+/CD163+ ratio	
Median (IQR)	1.8 (1.23–3.23)
PD-L1 CPS	
< 1 (%)	20 (71.4)
≥ 1 (%)	8 (28.6)

There was a significant association between histology subtype (ccRCC vs nccRCC) and number of CD80+, CD163+ cells and their ratio; particularly, a higher value of ratio between CD80+ and CD163+ cells was observed in nccRCC samples (median 1.60 vs 13.70; *p* = 0.003). A statistically significant association was also found between sarcomatoid features and number of CD4+ cells and ratio between CD4+ and CD8+ immune cells; numerically higher values of CD4+ cells (median 44 vs 16; *p* = 0.017) and higher CD4+/CD8+ ratio (median 2.10 vs 1.40; *p* = 0.016) were observed in tumor samples with sarcomatoid features. Additionally, patients with metastatic stage at initial diagnosis of RCC were significantly associated with higher expression of CD163+ ICs compared to patients without metastatic stage at diagnosis (median 53 vs 34; *p* = 0.017). No significant association was found between the IMDC score and each of the examined markers. Comparison between TME markers and clinicopathological features was reported in Table 3.

**Table 3:** Comparison between evaluated IHC TME markers and clinicopathological features of enrolled patients

TME marker	Histology		Sarcomatoid		IMDC score			MTS at diagnosis	
	ccRCC	nccRCC	Yes	No	Fav	Int	Poor	Yes	No
CD4+ IC									
Median	24	19	44	16	24	16	53	36	17
	*p value* = *0.39*		***p value* = *0.017***		*p value* = *0.064*			*p value* = *0.208*	
CD8+ IC									
Median	19	14	21	15	20	12	22	21	12
	*p value* = *0.59*		*p value* = *0.460*		*p value* = *0.255*			*p value* = *0.272*	
CD4+/CD8+ ratio									
Median	1.85	2.10	2.10	1.40	1.20	1.75	2.10	1.70	1.90
> 1 (%)	9 (37.5)	3 (75)	6 (100)	12 (54.5)	2 (66.6)	13 (59)	3 (100)	5 (71.4)	13 (61.9)
≤ 1 (%)	15 (62.5)	1 (25)	0	10 (45.5)	1 (37.3)	9 (41)	0	2 (28.6)	8 (38.1)
	*p value* = *0.874*		***p value* = *0.016***		*p value* = *0.516*			*p value* = *0.836*	
CD80+ IC									
Median	84	128	80	88	78	91	68	95	81
	***p value* = *0.035***		*p value* = *0.604*		*p value* = *0.433*			*p value* = *0.296*	
CD163+ IC									
Median	45	13	48	44	22	47	43	53	34
	***p value* = *0.035***		*p value* = *0.565*		*p value* = *0.225*			***p value* = *0.017***	
CD80+/CD163+ ratio									
Median	1.60	13.70	1.25	2.20	4.10	1.70	1.20	1.30	1.8
	***p value* = *0.003***		*p value* = *0.170*		*p value* = *0.129*			*p value* = *0.228*	
PD-L1 CPS									
< 1 (%)	17 (70.8)	3 (75)	4 (66.6)	16 (72.7)	3 (100)	16 (72.7)	1 (33.4)	4 (57.1)	15 (71.4)
≥ 1 (%)	7 (29.2)	1 (25)	2 (33.4)	6 (27.3)	0	6 (27.3)	2 (66.6)	3 (42.9)	6 (28.6)
	*p value* = *1*		*p value* = *0.764*		*p value* = *0.118*			*p value* = *0.917*	

Italics have been used to write the *p*-value

Statistically significant associations were highlighted using bold and italics for the *p*-value

*MTS* metastatic stage, *ccRCC* clear cell *RCC*, *nccRCC* non-clear cell RCC

### TME assessment and activity of first line immunotherapy-based combinations

Among the whole population included in our study, first-line immunotherapy-based combination treatment showed ORR of 42.9% (3.6% complete response, 39.3% partial response); stable disease as best overall response rate was achieved in 6 patients (21.4%), whereas 10 patients (35.7%) rapidly developed disease progression. The ORR was 33.3% and 50% in patients who received dual immune checkpoint blockade (nivolumab plus ipilimumab) and ICI+TKI combination (pembrolizumab plus axitinib or nivolumab plus cabozantinib), respectively.

According baseline clinical and disease characteristics (performance status, histology, presence of sarcomatoid features, previous nephrectomy, IMDC risk score, metastatic stage at initial diagnosis, type of combination treatment, type of tumor sample), no significant difference was observed between responder (defined as those who achieved a complete or partial response as best overall response) and non-responder patients (Table S1, Supplementary Information).

Responder patients were significantly associated with lower number of CD163+ ICs (median 28 vs 47; *p* = 0.049). No significant difference between responder and non-responder patients was observed according to any other TME marker. According ROCs based on presence of objective response (complete or partial response) to first-line treatment, no optimal cut-off value could be identified for each considered TME marker. Main results of this comparison were reported in Table 4.

**Table 4:** Comparison between expression of selected TME markers and response to first-line treatment in the entire population

TME marker	Responders (*N* = 12)	Non-responders (*N* = 16)	*p* value
CD4+ IC			
Median (IQR)	20 (13–40)	26 (10–41)	0.732
CD8+ IC			
Median (IQR)	16 (7–27)	19 (7–24)	0.909
CD4+/CD8+ ratio			
Median (IQR)	1.75 (0.85–2.73)	1.9 (0.73–3.15)	0.909
> 1 (%)	8 (66.7)	10 (62.5)	
≤ 1 (%)	4 (33.3)	6 (37.5)	1
CD80+ IC			
Median (IQR)	82 (65–92)	93 (71–120)	0.189
CD163+ IC			
Median (IQR)	28 (21–54)	47 (36–56)	**0.049**
CD80+/CD163+ ratio			
Median (IQR)	2.05 (1.2–4.25)	1.7 (1.35–2.58)	0.732
PD-L1 CPS			
< 1 (%)	10 (83.3)	10 (62.5)	
≥ 1 (%)	2 (16.7)	6 (37.5)	0.401

Statistically significant associations were highlighted using bold for the *p*-value

### TME assessment and efficacy of first line immunotherapy-based combinations

No statistically significant difference was found between patients who experienced disease progression within 12 months of first-line treatment with ICI compared to those who progressed after 12 months, according to selected TME markers. According ROCs based on presence of PFS greater than 12 months to first-line treatment, no optimal cut-off value could be identified for each considered TME marker.

For each TME marker, patients were classified into high (≥ median) and low (< median) groups.

**Fig. 1:**
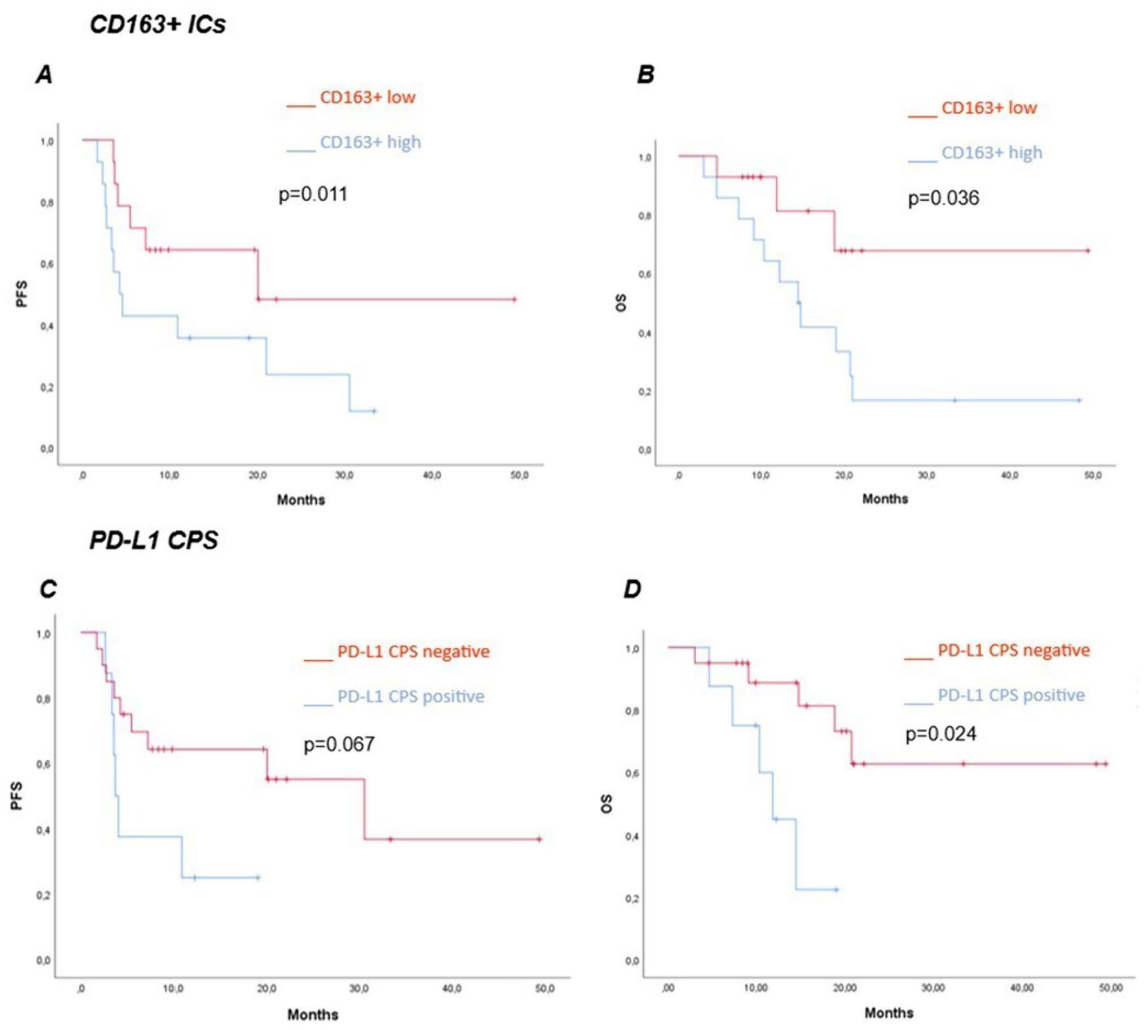
PFS and OS with first line immunotherapy-based combination according to count of CD163+ ICs (**A**, **B**) and PD-L1 CPS (**C**, **D**)

**Fig. 2:**
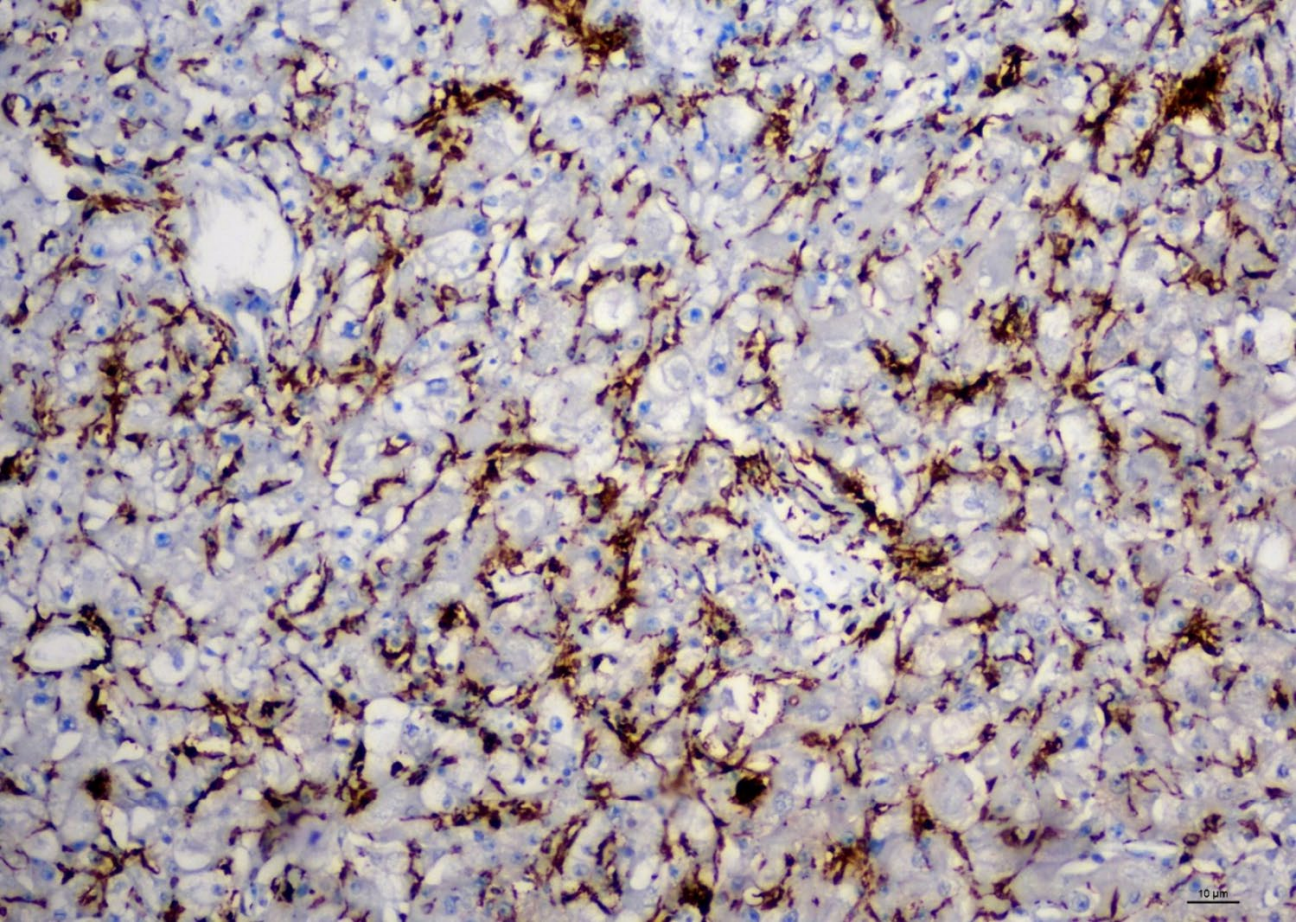
Immunohistochemical analysis of CD163 expression of macrophages in RCC samples with high expression (10× magnification, scale bar = 10 μm)

Lower expression of CD163+ ICs was significantly associated with better PFS (median PFS 20.0 vs 4.7 months; HR 0.22 95% CI 0.06–0.81 log rank *p* = 0.011) and OS (median OS NR vs 14.4 months; HR 0.28 95% CI 0.08–0.98 log rank *p* = 0.036) (Fig. 1A, B). Patients with PD-L1 CPS negative expression had significantly longer OS (median OS NR vs 11.8 months; HR 0.20 95% CI 0.05–0.80 log rank *p* = 0.024) compared to those whose tumor express PD-L1 CPS ≥ 1. Furthermore, PFS was longer in PD-L1 CPS negative patients, although this difference was not statistically significant (median PFS 30.5 vs 3.7 months; HR 0.37 95% CI 0.12–1.19 log rank *p* = 0.067) (Figs. 1C, D and 2). There were no significant differences in PFS or OS according to count of CD4+, CD8+, CD80+ cells or CD4+/CD8+ ratio.

## Discussion

In our experience, we provide a pathological characterization of TME of tumor blocks from patients treated with first line ICI-based treatment using multiple IHC assay. In case of metachronous presentation, only cases with histological material from no more than 3 months before the diagnosis of metastatic stage were included, in order to minimize any discrepancies in the representation of TME of metastatic disease as much as possible [17].

The results derived from the IHC analysis of our sample are quite similar to those previously reported in literature, particularly for ratio between CD4+ and CD8+ cells [18]; only a third of patients presented PD-L1 CPS above 1. Tumors without clear cell component were associated with higher ratio between CD80+ and CD163+ immune cells, reflecting distinct macrophage population between ccRCCs and nccRCCs [19]. Higher number of CD4+ T cells was observed in patients whose tumor expressed sarcomatoid dedifferentiation, that is commonly associated with an immune-inflamed phenotype characterized by immune activation, upregulation of antigen presentation genes and, finally, good clinical response to immune checkpoint blockade [20]. This finding underlines the controversial role of CD4+ immune cells in cancer immune response: various subsets of CD4+ ICs can either suppress (T helper 2 CD4+ and T regulatory CD4+ cells) or promote (T helper 1 CD4+ cells and CD4+ cytotoxic lymphocytes) antitumor immunity modulating cytotoxic T cell responses [21]. In patients whose RCC was metastatic at initial diagnosis, higher value of CD163+ macrophage was observed.

An important role of CD163+ cell line was also found in further analysis performed in relation to activity and efficacy of first line treatment. A significant difference was observed between patients who achieved a partial or complete disease response compared to those who did not respond. In particular, a lower CD163+ macrophage count was found in tumor samples of patients who responded to immunotherapy. Similarly, patients with lower CD163+ cell count experienced better clinical outcome, developing significantly longer PFS and OS with combination treatment based on immune checkpoint blocker compared to group of patients who had higher infiltration of CD163+ macrophages.

Notably, high infiltration of CD163+ TAMs in microenvironment has been reported to be associated with poor prognosis in RCC [22], promoting cancer cell proliferation through direct cell–cell interactions [23]; this concept was already well known in the pre immunotherapy era of renal carcinoma [24]. Tumor cell population can also express CD163, and detection of high number of CD163+ tumor cells is associated with poor prognosis and shorter survival in lung adenocarcinoma [25]. CD163 is the high affinity scavenger receptor for hemoglobin–haptoglobin complex and is commonly considered a typical marker of M2 macrophages, while its expression is generally low in monocytes. M2 macrophage subpopulation represents the main type of TAM and is involved in tumor development and progression; production of anti-inflammatory cytokines, such as IL-4, IL-10 and IL-13 (produced by T helper 2 cells), can induce polarization of TAMs towards M2 phenotype and is associated with the expression of CD163 [15]. This macrophage subpopulation plays a critical role in immune evasion, expressing anti-inflammatory elements (IL-10 and TGF-β, arginase-1) [26]. Their action leads to downregulation of the anti-tumor response orchestrated by the T helper 1-mediated adaptive immunity, promoting the development of the less effective T helper 2 mediated immune response [27]. As a result, an innate or acquired resistance to immune checkpoint blockade can be induced by these modifications in TME [28]. Furthermore, high serum levels of soluble CD163 were found to be associated with worse clinical outcome in metastatic RCC [29]; its measurement during treatment with anti PD-1 therapy seems to play a predictive role of effectiveness in patients with advanced cutaneous melanoma [30]. Our study confirms the role of a high CD163+ macrophage count in TME in a population of patients who received a first-line combination treatment with ICIs: this marker can identify a subgroup of patients with poor prognosis, with limited clinical benefit from use of immunotherapy compared to patients with lower count. Novel approaches, including use of anti CD163 antibody (such as OR2805, first-in-class of this drug group), could reduce immunosuppression mediated by M2c macrophages and enhance T cell activity, reprogramming M2-like macrophages to an antitumor M1-like phenotype [31]. Ongoing clinical trials are evaluating safety and activity of OR2805 alone or in combination with other drugs (ICIs and/or chemotherapy) in multiple solid tumors [32].

The utility of PD-L1 determination remains a controversial issue in advanced RCC. Historically, high expression of PD-L1 was considered a negative prognostic factor and was associated with shorter overall survival (especially in ccRCC) [33, 34]. The predictive value of PD-L1 in patients treated with immunotherapy was assessed in several meta-analyses that included main pivotal trials of first-line combinations. PD-L1 expression was a reliable biomarker only for PFS [35], whereas ICI-based treatments showed to improve OS compared to sunitinib in all patients, regardless of PD-L1 positivity [36]. In addition, PD-L1 expression was evaluated using different antibodies (22C3, 28-8, SP263, and SP142), platforms (Ventana and Dako) and scoring systems in various clinical trials, generating different results [37]. These findings underlined the limited role of PD-L1 as predictive biomarker. In our analysis, we used CPS to determine PD-L1 expression, evaluating all PD-L1 positive cells (tumor cells, lymphocytes and macrophages) in tumor microenvironment. Patients whose tumor expressed PD-L1 had shorter OS compared to patients with PD-L1 negative RCC; however, only a small number of examined tumor samples (8 out of 28) had PD-L1 CPS above 1. These results seem to underline a possible negative prognostic role of PD-L1 positivity also in patients who received ICI-based treatments, although this is not so clear from current literature evidence. Indirect comparisons from CheckMate 214 showed numerically shorter median OS in patients treated with nivolumab plus ipilimumab and PD-L1 CPS positive tumor compared to patients whose tumor did not express PD-L1 [38]. Nevertheless, our results should be interpreted in relation to the small sample size.

The present study had several limitations that should be underlined. Particularly, the retrospective nature and the small sample size remarkably reduce the power of statistical analysis and do not allow to draw definitive conclusions. Due to the limited number of patients, we did not distinguish patients on the basis of the type of combination therapy (dual ICI or ICI in combination with TKI) and not perform separate statistical analyses. Additionally, no optimal cut-off value for each TME marker could be identified, and patients were dichotomized on the basis of the median value of cell count. A small proportion of patients had nccRCC: due to the small overall sample size, this has contributed to reducing the homogeneity of our study population. Finally, although the majority of examined specimens consisted of the primary tumor, in 14.3% of cases histological material from metastatic sites was analyzed. Several findings suggest possible differences in the composition of TME between primary tumor and metastasis; however, these discrepancies seem to be limited to some populations of immune cells (such as mature dendritic cells), while greater concordance is evident for CD8+ lymphocytes and natural killer cells [39, 40].

The main strength of our study is the effort to provide a simple characterization of TME by IHC assay and to highlight the key role played by TME cell population in guiding prognosis and antitumor response mediated by first-line treatment with ICIs in RCC patients. Notably, IHC could represent a simple, reproducible and cost-effective method to evaluate TME composition. In our analysis, higher count of specific cell lines (such as CD163+ TAMs) appears to be associated with worse clinical outcome in patients who were treated with first line immunotherapy-based combination therapy.

Further analyses, based on prospective, comparative design and large population of patients, are needed to validate role of TME biomarkers in RCC patients treated with first-line immunotherapy.

## Conclusions

Despite main limitations, such as retrospective nature and small sample size, our study focuses on the importance of TME in RCC, whose IHC assessment could help to identify a subgroup of patients with worse clinical outcome after receiving immune checkpoint blockers. Specifically, a high CD163+ cell count seems to be associated with poor response and short survival in patients who were treated with first-line ICI-based combination for advanced RCC. Prospective trials, with a well-designed comparative design, are needed to clarify role of TME-related pathological biomarkers.

### Supplementary Information

Below is the link to the electronic supplementary material.

Supplementary file1 (PDF 515 KB)

## Data Availability

The datasets generated during and/or analyzed during the current study are available from the corresponding author on reasonable request.

## Abbreviations

TME: Tumor microenvironment
ICI: Immune checkpoint inhibitor
RCC: Renal cell carcinoma
IHC: Immunohistochemistry
CD: Cluster of differentiation
PFS: Progression-free survival
OS: Overall survival
NR: Not reached
PD-L1: Programmed death-ligand 1
CPS: Combined positive score

## References

[1] Powles T (2021). ESMO clinical practice guideline update on the use of immunotherapy in early stage and advanced renal cell carcinoma. Ann Oncol.

[2] Motzer RJ (2022). Conditional survival and long-term efficacy with nivolumab plus ipilimumab versus sunitinib in patients with advanced renal cell carcinoma. Cancer.

[3] Plimack ER (2023). Pembrolizumab plus axitinib versus sunitinib as first-line treatment of advanced renal cell carcinoma: 43-month follow-up of the phase 3 KEYNOTE-426 study. Eur Urol.

[4] Burotto M (2023). Nivolumab plus cabozantinib vs sunitinib for first-line treatment of advanced renal cell carcinoma (aRCC): 3-year follow-up from the phase 3 CheckMate 9ER trial. J Clin Oncol.

[5] Motzer RJ (2023). Final prespecified overall survival (OS) analysis of CLEAR: 4-year follow-up of lenvatinib plus pembrolizumab (L+P) vs sunitinib (S) in patients (pts) with advanced renal cell carcinoma (aRCC). J Clin Oncol.

[6] Lalani AA (2022). Evolving landscape of first-line combination therapy in advanced renal cancer: a systematic review. Ther Adv Med Oncol.

[7] Moreira M (2020). Resistance to cancer immunotherapy in metastatic renal cell carcinoma. Cancer Drug Resist.

[8] Rosellini M (2023). Prognostic and predictive biomarkers for immunotherapy in advanced renal cell carcinoma. Nat Rev Urol.

[9] Saliby RM (2024). Update on biomarkers in renal cell carcinoma. Am Soc Clin Oncol Educ Book.

[10] Wu T, Dai Y (2017). Tumor microenvironment and therapeutic response. Cancer Lett.

[11] Giraldo NA (2015). Orchestration and prognostic significance of immune checkpoints in the microenvironment of primary and metastatic renal cell cancer. Clin Cancer Res.

[12] Monjaras-Avila CU (2023). The tumor immune microenvironment in clear cell renal cell carcinoma. Int J Mol Sci.

[13] Kalra J, Baker J (2017). Multiplex immunohistochemistry for mapping the tumor microenvironment. Methods Mol Biol.

[14] Kim JH (2022). Clinical implications of the tumor microenvironment using multiplexed immunohistochemistry in patients with advanced or metastatic renal cell carcinoma treated with nivolumab plus ipilimumab. Front Oncol.

[15] Skytthe MK, Graversen JH, Moestrup SK (2020). Targeting of CD163. Int J Mol Sci.

[16] Chen Y (2019). Tumor-associated macrophages: an accomplice in solid tumor progression. J Biomed Sci.

[17] Fujita K (2022). The association of tumor immune microenvironment of the primary lesion with time to metastasis in patients with renal cell carcinoma: a retrospective analysis. Cancers.

[18] Rebuzzi SE (2023). Characterization of tumor and immune tumor microenvironment of primary tumors and metastatic sites in advanced renal cell carcinoma patients based on response to nivolumab immunotherapy: preliminary results from the meet-URO 18 study. Cancers.

[19] Govindarajan A (2023). Characterization of papillary and clear cell renal cell carcinoma through imaging mass cytometry reveals distinct immunologic profiles. Front Immunol.

[20] Bakouny Z (2021). Integrative molecular characterization of sarcomatoid and rhabdoid renal cell carcinoma. Nat Commun.

[21] Ben Khelil M (2022). Harnessing antitumor CD4. Cancers.

[22] Ma C (2018). CD163-positive cancer cells are potentially associated with high malignant potential in clear cell renal cell carcinoma. Med Mol Morphol.

[23] Zhu D (2023). Combining expression of RNF43 and infiltration level of CD163. Cancer Med.

[24] Komohara Y (2011). Macrophage infiltration and its prognostic relevance in clear cell renal cell carcinoma. Cancer Sci.

[25] Matsubara E (2021). CD163-positive cancer cells are a predictor of a worse clinical course in lung adenocarcinoma. Pathol Int.

[26] Shapouri-Moghaddam A (2018). Macrophage plasticity, polarization, and function in health and disease. J Cell Physiol.

[27] Chen S (2023). Macrophages in immunoregulation and therapeutics. Signal Transduct Target Ther.

[28] Ceci C (2020). Targeting tumor-associated macrophages to increase the efficacy of immune checkpoint inhibitors: a glimpse into novel therapeutic approaches for metastatic melanoma. Cancers.

[29] Lauridsen KM (2023). Soluble CD163: a novel independent prognostic biomarker in patients with metastatic renal cell carcinoma. Cancer Immunol Immunother.

[30] Fujimura T (2018). Serum level of soluble CD163 may be a predictive marker of the effectiveness of nivolumab in patients with advanced cutaneous melanoma. Front Oncol.

[31] Probst P (2021). 271 development of OR2805, an anti-CD163 antibody derived from an elite responder to checkpoint inhibitor therapy that relieves immunosuppression caused by M2c macrophages. J Immunother Cancer.

[32] Tolcher AW (2022). A phase 1/2 dose escalation/expansion study of OR2805 alone or in combination in subjects with advanced solid tumors. J Clin Oncol.

[33] Iacovelli R (2016). Prognostic role of PD-L1 expression in renal cell carcinoma. A systematic review and meta-analysis. Target Oncol.

[34] Wang Z (2018). Prognostic and clinicopathological significance of PD-L1 in patients with renal cell carcinoma: a meta-analysis based on 1863 individuals. Clin Exp Med.

[35] Mori K (2021). The predictive value of programmed death ligand 1 in patients with metastatic renal cell carcinoma treated with immune-checkpoint inhibitors: a systematic review and meta-analysis. Eur Urol.

[36] Carretero-González A (2020). The value of PD-L1 expression as predictive biomarker in metastatic renal cell carcinoma patients: a meta-analysis of randomized clinical trials. Cancers.

[37] Vranic S, Gatalica Z (2023). PD-L1 testing by immunohistochemistry in immuno-oncology. Biomol Biomed.

[38] Motzer RJ (2022). Biomarker analysis from CheckMate 214: nivolumab plus ipilimumab versus sunitinib in renal cell carcinoma. J Immunother Cancer.

[39] Remark R (2013). Characteristics and clinical impacts of the immune environments in colorectal and renal cell carcinoma lung metastases: influence of tumor origin. Clin Cancer Res.

[40] Baine MK (2015). Characterization of tumor infiltrating lymphocytes in paired primary and metastatic renal cell carcinoma specimens. Oncotarget.

