# Supplementary material for: Tumor microenvironment and clinical efficacy of first line immunotherapy-based combinations in metastatic renal cell carcinoma
Source: Med Oncol. 2024 May 13;41(6):150. doi: 10.1007/s12032-024-02370-0 (PMC11090963; doi:10.1007/s12032-024-02370-0)
Supplement: Supplementary file 1 — Supplementary file1 (PDF 515 KB) [file 12032_2024_2370_MOESM1_ESM.pdf]

SUPPLEMENT TO:

Tumor microenvironment and clinical efficacy of first line immunotherapy-based combinations in metastatic renal cell carcinoma

Medical Oncology 2024

Enrico Sammarco[1,], Martina Rossetti[2], Alessia Salfi[3], Adele Bonato[3], Paolo Viacava[4], Gianluca Masi[3,5], Luca Galli[3] and Pinuccia Faviana[2]*

1.Medical Oncology Unit, Livorno Hospital, Azienda Toscana Nord Ovest, Livorno, Italy

2. Department of Surgical, Medical, Molecular Pathology and Critical Area, University of Pisa, Pisa, Italy

3.Medical Oncology Unit 2, Santa Chiara Hospital, Azienda Ospedaliero-Universitaria Pisana, Pisa, Italy

4.Pathology Unit, Livorno Hospital, Azienda Toscana Nord Ovest, Livorno, Italy

5. Department of Translational Research and New Technologies in Medicine and Surgery, University of Pisa, Pisa, Italy

**corresponding author (enricosammarco1992@gmail.com)*

This appendix has been provided by the authors to give readers additional information about the work.

List of Contents

Table S1. Comparison between baseline characteristic and response to first-line treatment 2

Characteristic	*Responders (N=12)*	*Non-responders (N=16)*	*p-value*
**ECOG performance status**			
0	8 (66.7)	10 (62.5)	
1	4 (33.3)	4 (25.0)	
2	0	2 (12.5)	0.692
**Histology**			
ccRCC, n (%)	10 (83.3)	14 (87.5)	1
nccRCC, n (%)	2 (16.7)	2 (12.5)	
**Sarcomatoid features**			
Yes	3 (25.0)	3 (18.8)	1
No	9 (75.0)	13 (81.2)	
**Nephrectomy**			
Yes	10 (83.3)	14 (87.5)	1
No	2 (16.7)	2 (12.5)	
**Metastatic stage at initial diagnosis**			
Yes	2 (16.7)	5 (31.2)	
No	10 (83.3)	11 (68.8)	0.662
**IMDC score**			
Favorable	3 (25.0)	0	
Intermediate	7 (58.3)	15 (93.8)	
Poor	2 (16.7)	1 (6.2)	0.068
**Type of 1st line combination treatment**			
Dual ICI	4 (33.3)	8 (50.0)	
ICI+TKI	8 (66.7)	8 (50.0)	0.459
**Type of tumor sample**			
Primary tumor	10 (83.3)	14 (87.5)	1
Metastasis	2 (16.7)	2 (12.5)	

Table S1. *Comparison between baseline characteristic and response to first-line treatment in the entire population*
